# Transcriptomics in solanaceous crop improvement: advances and opportunities

**DOI:** 10.3389/fpls.2025.1750317

**Published:** 2026-01-16

**Authors:** Xinlong Qiao, Bijiao Jian, Jiatong Qiu, Jagmohan Singh, Ishveen Kaur, Ronglin Hao, Jasdeep Singh, Lovepreet Singh, Huijun Zhang, Xueren Yin, Xiang Li, Gurleen Kaur, Haisu Li

**Affiliations:** 1 School of Horticulture, Anhui Agricultural University, Hefei, China; 2 Department of Plant Pathology, CCS Haryana Agricultural University, Hisar, India; 3 Department of Horticulture, Virginia Polytechnic Institute and State University, Blacksburg, VA, United States; 4 Horticultural Sciences Department, University of Florida, Gainesville, FL, United States; 5 Department of Plant and Soil Sciences, Mississippi State University, Starkville, MS, United States; 6 School of Life Science, Huaibei Normal University, Huaibei, Anhui, China

**Keywords:** crop quality, gene regulation, microarray, RNA-seq, solanaceous, transcriptomics

## Abstract

The solanaceae family includes several economically important crops. Tomato (*Solanum lycopersicum*), potato (*S. tuberosum*), eggplant (*S. melongena*), and pepper (*Capsicum annuum*) are among the most consumed vegetables worldwide. Over the past few decades, consumers have often expressed dissatisfaction with declining fruit quality, especially flavor, while growers’ profits remain tied primarily to yield and stress resistance. Thus, reconciling these divergent demands provides a clear roadmap for improving solanaceous crops. To completely understand molecular mechanisms underlying biological processes associated with fruit quality, yield, and stresses, there is a need to focus on gene expression at the RNA level. With the availability of next-generation sequencing (NGS) technologies, transcriptomics platforms have helped to improve our knowledge about RNA-based gene regulatory networks. The current review discusses the recent literature on transcriptomics techniques and their progress status in major solanaceae crops across important agronomic traits. In addition to the current improvements, we have also discussed future perspectives of transcriptomics in genetic engineering.

## 1 Introduction

Increasing temperatures and shrinking water availability in major fruit and vegetable production regions threatens the availability of nutritious food globally (Cammarano et al., 2022; Wang et al., 2017). Among horticultural crops, the Solanaceae family represents a strategic target for genetic improvement due to its nutritional importance and genetic diversity (Kaur et al., 2023). With the global population increasing to an estimated 9.7 billion by 2050, it is necessary to accelerate genetic gain in highly nutritious Solanaceae crops (Keating et al., 2014; Sandhu et al., 2022; Kantar et al., 2025).

The Solanaceae family encompasses ~100 genera and 2,500 species (Olmstead et al., 2008) among which tomato (*S. lycopersicum*), pepper (*C. annuum* L.), potato (*S. tuberosum*), and eggplant (*S. melongena*) are significant contributors to nutrition availability. Furthermore, secondary metabolites from these crops are used in pharmaceuticals, flavoring agents, and fragrances (Fiesel et al., 2022). However, these solanaceous crops face a critical trade-off between consumer preferences and commercial production objectives. While consumers prioritize fruit quality attributes like flavor, freshness, and appearance (Zhang et al., 2023), enhanced tolerance to heat, drought, and biotic stresses is of utmost importance to farmers. This diversity in trait priorities necessitates a multi-trait breeding strategy that simultaneously addresses yield, stress resilience and fruit quality traits (Kaur et al., 2023; Singh et al., 2025).

**Figure 1:**
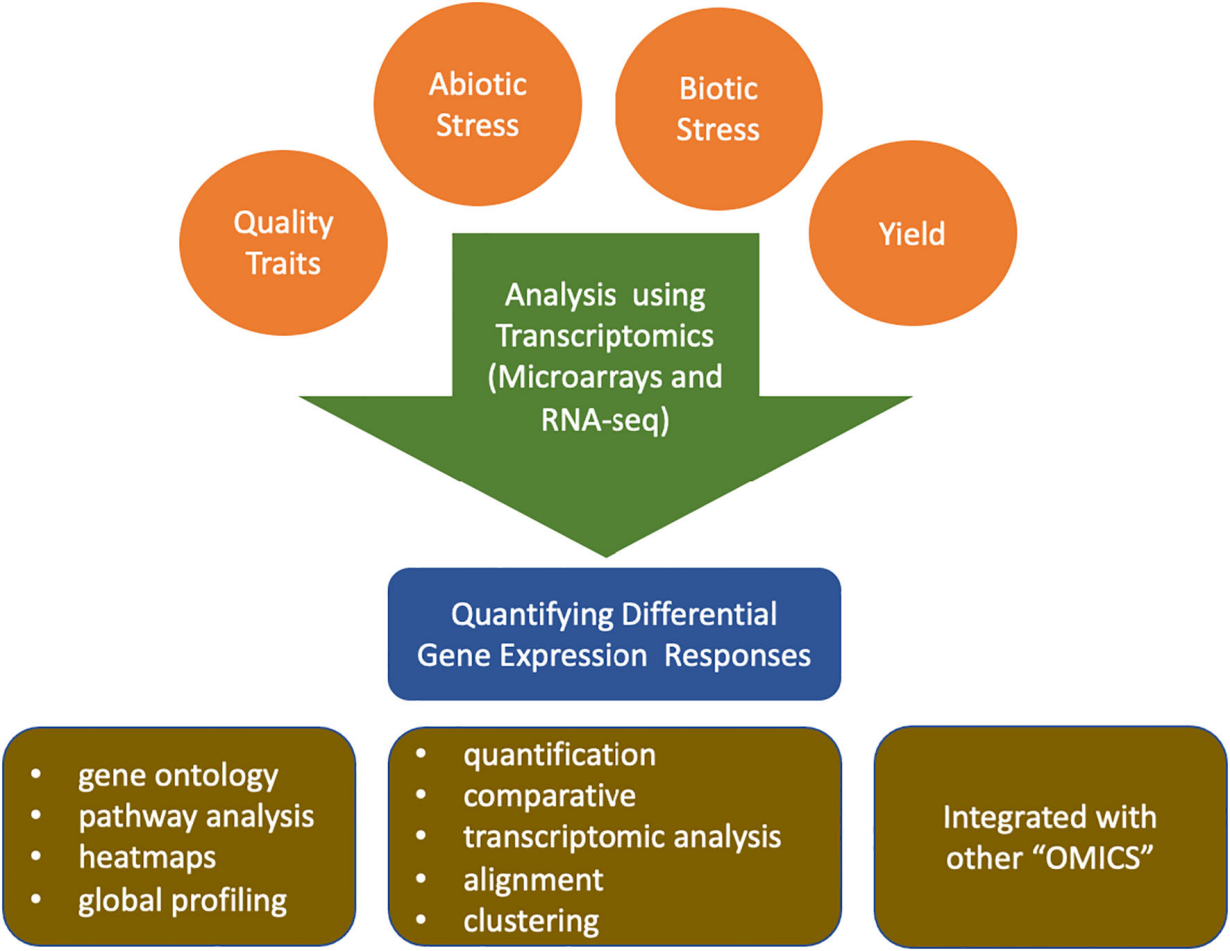
An overview of the utilization of transcriptomics techniques in economically important traits in solanaceous crops.

To bridge the gap between consumer demands and production constraints while adapting to climate change, solanaceous crop breeding can exploit the knowledge derived from molecular and genetic mechanisms of tolerance to heat, drought, biotic stresses and flavor. Next-generation sequencing (NGS) and transcriptomics have enhanced our ability to characterize genome-wide expression patterns to construct functional gene regulatory networks for these complex traits. Transcriptomic studies have successfully identified candidate genes conferring resistance to biotic stresses (Meng et al., 2021) abiotic stresses (Altaf et al., 2022), enhanced flavor compounds (Gao et al., 2019), improved post-harvest quality (Cruz-Mendívil et al., 2015; Popovsky-Sarid et al., 2017; Wahrenburg et al., 2021) and nutritional content (Kim et al., 2014) (**Figure 1**). In solanaceous crops, transcriptomic approaches are particularly valuable for dissecting complex, polygenic traits where phenotypic selection alone proves insufficient. Developmental processes and stress responses are governed by intricate signaling cascades that operate across temporal and spatial scales, with coordinated gene expression patterns ultimately determining phenotypic outcomes. However, existing reviews have focused on individual species or single trait categories, while a cross-species, multi-trait synthesis examining transcriptomic insights across stress tolerance, yield, and quality traits remains absent in solanaceous crops. This review paper addresses the aforementioned knowledge gap and provides a snapshot of recent advances in transcriptomics of solanaceous crop improvement emphasizing translational insights for multi-trait breeding strategies.

## 2 Timeline of the development of transcriptomics technology in Solanaceae crops

**Figure 2:**
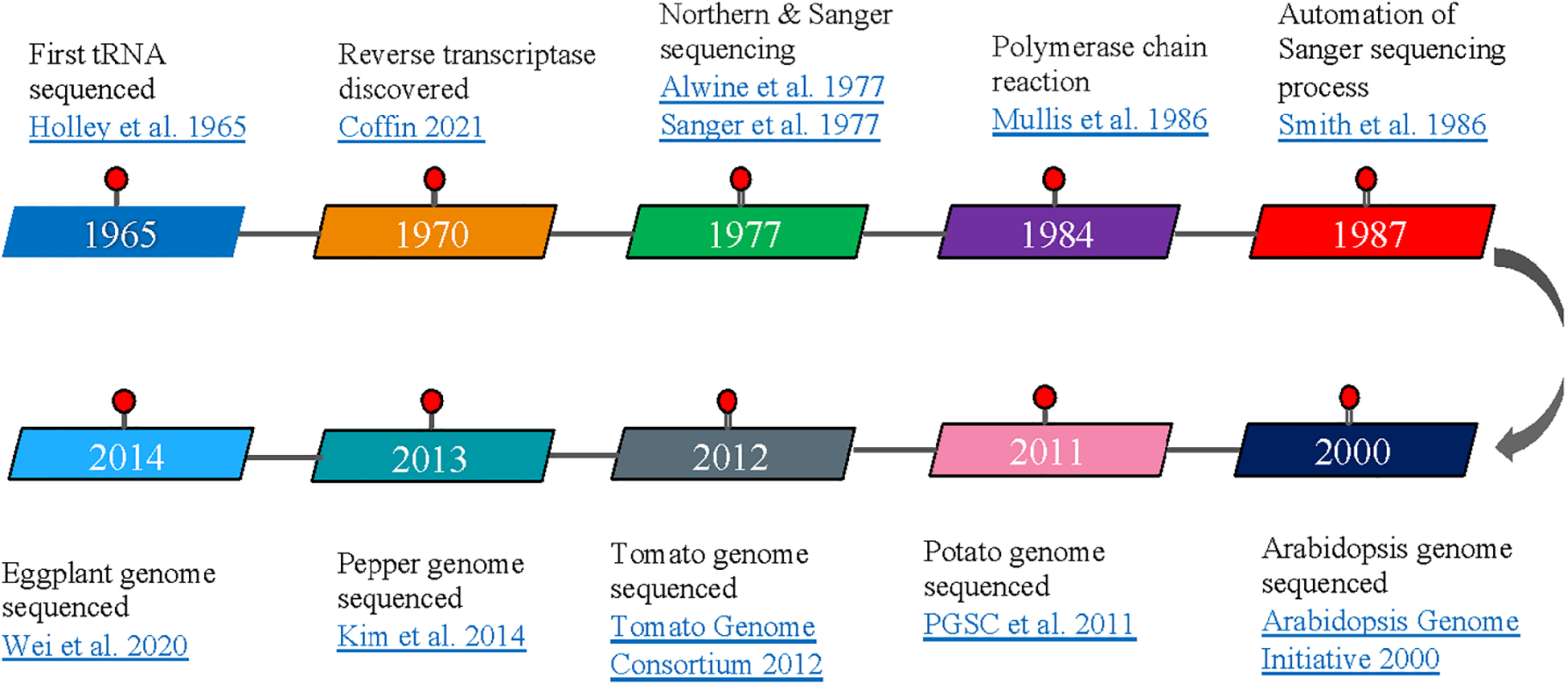
Timeline of the major advancements contributing to transcriptomics emergence as a tool in solanaceous crop improvement.

The integration of transcriptomics with genomic resources has transformed candidate gene identification in Solanaceae breeding. By integrating gene expression profiles with genomic regions identified through genome-wide association studies and quantitative trait loci (QTL) mapping, breeders can prioritize causal genes from among multiple candidates and develop functional markers that capture allelic variation affecting expression levels. This expression quantitative trait loci (eQTL) approach enables more precise marker-assisted selection and accelerates the validation of genes controlling complex traits. Multiple technological platforms have emerged to interrogate transcriptomes, each offering distinct advantages for specific applications. These range from hybridization-based microarrays to high-throughput RNA sequencing (RNA-seq), single-cell transcriptomics, and specialized methods for profiling small regulatory RNAs. We review these approaches sequentially (**Figure 2**), emphasizing their applications in solanaceous crop improvement and the insights each technology has provided for breeding programs.

Early transcriptomic approaches were constrained by their single-gene focus. Northern blotting enabled detection and quantification of specific transcripts through gel separation and probe hybridization (Alwine et al., 1977), while reverse transcription PCR and cDNA library construction allowed targeted gene expression analysis (Mullis et al., 1986). However, these methods could not capture the genome-wide expression dynamics necessary for understanding complex regulatory networks underlying multi-trait phenotypes.

The need for simultaneous profiling of thousands of genes drove the development of microarray platforms in the late 1990s. Microarrays operate by hybridizing fluorescently labeled RNA samples to gene-specific probes arrayed at known positions on solid support. Early platforms utilized cDNA probes derived from expressed sequence tags (ESTs), while later iterations employed synthesized oligonucleotides; either short probes with multiple sequences per gene (25-mers on Affymetrix chips) or longer probes with single or few representatives (>60-mers) (Yamada et al., 2003). In Solanaceae, microarrays enabled the first genome-scale expression studies, including identification of ripening-related transcripts in tomato (Karlova et al., 2011). Despite these advances, microarrays suffered from substantial limitations: 40-60% of genes showed no detectable expression due to high background noise, expression distributions were highly skewed toward low-abundance transcripts, and probe design required prior sequence knowledge (Klebanov and Yakovlev, 2007; Zhao et al., 2014).

To address microarray limitations while maintaining high throughput, tag-based sequencing methods emerged, including serial analysis of gene expression (Matsumura et al., 1999), cap analysis of gene expression (Agarwal et al., 2014), and massively parallel signature sequencing (Meyers et al., 2004). These approaches sequenced short tags derived from defined transcript positions, eliminating hybridization bias and enabling discovery of novel transcripts without prior sequence knowledge. However, short tag lengths (typically 14–21 nucleotides) prevented discrimination of alternative splice isoforms and paralogs with high sequence similarity highlighting critical limitations for studying gene families in polyploid crops like potato, hence ultimately restricting widespread adoption (Wang et al., 2009).

RNA-seq fundamentally transformed transcriptome profiling by replacing hybridization-based detection with direct sequencing and digital quantification of cDNA molecules derived from RNA samples (Wang et al., 2009). This approach eliminates probe design requirements and hybridization biases, enabling unbiased detection of all transcripts including protein-coding mRNAs and diverse non-coding RNAs (lncRNA, miRNA, circRNA, rRNA, and tRNA) without prior sequence knowledge. By counting sequence reads aligned to genomic or transcript references, RNA-seq provides absolute quantification across a dynamic range exceeding five orders of magnitude, detecting both highly abundant transcripts and rare, lowly expressed genes that evade microarray detection. This sensitivity has proven particularly valuable in Solanaceae crops for capturing expression of biosynthetic pathway genes (often expressed at low levels) controlling flavor compound production in tomato and pepper (Gao et al., 2019).

Beyond improved sensitivity, RNA-seq enables three critical capabilities for dissecting complex traits: (1) Discovery of novel transcripts and unannotated genes through *de novo* assembly, expanding the functional repertoire of crop genomes (Liu et al., 2015); (2) Characterization of alternative splicing events and isoform diversity, revealing how single genes generate multiple protein products with distinct functions (Keller et al., 2017; Yang et al., 2020c); and (3) Detection of post-transcriptional modifications including RNA editing events that alter protein sequence (Zouine et al., 2014; Amiryousefi et al., 2018). In Solanaceae, these capabilities have been leveraged to identify candidate genes controlling fruit ripening and quality traits in tomato (Zhu et al., 2015), characterize the transcriptional architecture of heat and drought responses across multiple genotypes in pepper (Kim et al., 2024) and profile pathogen-responsive transcriptional networks in eggplant (Chen et al., 2018). The introduction of short-read sequencing platforms particularly Illumina’s sequencing-by-synthesis (Egan et al., 2012) and Ion Torrent’s semiconductor-based sequencing (Merriman et al., 2012) dramatically reduced per-sample costs from thousands to hundreds of dollars, democratizing access and enabling large-scale expression profiling across diverse genotypes, environments, and developmental stages.

Two technological advances have further expanded RNA-seq’s applications in crop genomics. First, long-read sequencing platforms including Pacific Biosciences (PacBio) isoform sequencing (Iso-Seq) and Oxford Nanopore direct RNA sequencing sequence full-length transcripts in single reads (>1–10 kb), eliminating the need for computational isoform reconstruction and enabling definitive characterization of alternative splicing, fusion transcripts, and allele-specific expression in heterozygous and polyploid genomes like potato. Second, single-cell RNA-seq (scRNA-seq) resolves transcriptomes of individual cells within heterogeneous tissues, revealing cell-type-specific expression programs that are masked in bulk tissue analyses (Stuart and Satija, 2019; Aldridge and Teichmann, 2020). In plants, scRNA-seq has begun elucidating cell-type-specific responses to developmental cues and environmental stresses (Seyfferth et al., 2021), though technical challenges remain: low transcript capture efficiency (5-20%), high per-cell costs limiting sample sizes, ambient RNA contamination from lysed cells, and critically for plant tissues with thick cell walls difficulties in generating high-quality single-cell suspensions. Emerging spatial transcriptomics technologies address this last limitation by preserving tissue architecture while profiling spatially resolved expression, offering promise for understanding fruit development and ripening processes where spatial gradients in gene expression determine quality traits.

## 3 Transcriptomics of fruit quality traits and regulatory networks

Solanaceous fruits are an essential source of phytochemicals and secondary metabolites that influence plant defense, fruit quality, UV protection, and signaling pathways (Carvalho Lemos et al., 2019). Transcriptomic approaches have proven particularly powerful for dissecting these complex biosynthetic pathways, where multi-enzyme cascades, tissue-specific expression, and coordinated regulation cannot be inferred from genome sequences alone. The quality traits of several solanaceous crops that have been examined using transcriptomics are listed in **Table 1**.

**Table 1:** Previous studies to understand quality traits in the solanaceae family using transcriptomics techniques.

Crop	Technique	Quality trait	Characteristics	References
eggplant	qRT-PCR	anthocyanin and CGA	transcript abundance in different tissues	(Docimo et al., 2016)
eggplant	RNA-seq	anthocyanin	screening for DEGs from transcriptomic data of eggplant peel during the transition from darkness to varying durations of light exposure	(Li et al., 2018a)
eggplant	RNA-seq	browning	potential DEGs associated with browning between the two eggplant cultivars were screened	(Liu et al., 2021b)
pepper	qRT-PCR	AsA	gene expression of biosynthetic genes is inversely related to AsA	(Alós et al., 2013)
pepper	RNA-seq	capsaicinoids	unigenes (transcripts) were assembled by RNA-seq for the mixture of placenta and pericarp of pungent pepper, and new candidate genes involved in capsaicinoid biosynthesis were predicted	(Liu et al., 2013)
pepper	RNA-seq	capsaicinoids and AsA	RNA-Seq was used to obtain transcriptomes of whole Serrano-type chili pepper fruits, and genes expression related to capsaicinoid and ascorbic acid biosynthesis were analyzed	(Martínez-López et al., 2014)
pepper	RNA-seq	quercetin and derivatives	biosynthetic pathway of quercetin and derivatives from sweet pepper fruits were analysis, and expression profile of genes was analysed	(Guevara et al., 2021)
pepper	RNA-seq	flavonoid, phenylalanine, tyrosine, tryptophan and phenylpropanoid	combined transcriptome and metabolome analysis showed that the genes and metabolites that responded to UV-C were mainly involved in flavonoid, phenylalanine, tyrosine, tryptophan and phenylpropanoid biosynthesis	(Ma et al., 2021a)
pepper	RNA-seq	fruit development and ripening(shape, size, ethylene and secondary metabolites biosynthesis)	transcriptomic analysis on immature stage and mature stage of a wild and a cultivated pepper variety, DEGs related to fruit ripening were futher screened	(Razo-Mendivil et al., 2021)
pepper	RNA-seq	ascorbate peroxidase	expression analysis of CaAPX genes by RNA-Seq at different stages of pepper fruit ripening	(González-Gordo et al., 2022)
pepper	multi-omics	flavonoids and carotenoids	DEGs were obtained and used for gene co-expression network analysis (WGCNA)	(Liu et al., 2020)
pepper	multi-omics	methyl jasmonate	model of methyl jasmonate-regulated postharvest chilling injury in green pepper fruits was studied by integrating transcriptomics, proteomics, and metabolomics	(Fu et al., 2022)
potato	microarray	carotenoids	differentially expressed microarray probes present within the major QTL for total tuber carotenoid content	(Campbell et al., 2014)
potato	multi-omics	soluble sugars	combined transcriptomic and proteomic analysis was conducted on potato tubers to investigate the mechanism of cold responses during postharvest storage	(Lin et al., 2019)
potato	multi-omics	anthocyanins	genomic data and the transcriptome data was to identify the crucial major genes that affect anthocyanin biosynthesis	(Tengkun et al., 2019)
potato	multi-omics	browning	postcutting browning were evaluated with transcriptomics and untargeted metabolomics to investigate the molecular mechanism	(Qiao et al., 2022)
tobacco	microarray	carotenoids	microarray was adapted for transcriptome comparison of tobacco leaves derived from three cultivated regions	(Lei et al., 2013)
tobacco	transcriptome	cadmium(cd)	DEGs obtained in the root and leaf samples of two Nicotiana species under the same Cd stress concentration	(Zhang et al., 2021c)
tomato	RNA-seq	chilling	DEGs responsed to chilling	(Zhang et al., 2016)
tomato	RNA-seq	malate accumulation	DEGs determined by RNA-seq in overexpression line	(Ye et al., 2017)
tomato	RNA-seq	ethylene synthesis, signal transduction and carotenoid biosynthesis	RNA-Seq was used to identify genes that were differentially expressed in 1-MCP-treated tomato fruits	(Bobokalonov et al., 2018)
tomato	RNA-seq	biosynthesis of terpenoids, alcohols and esters	characterize the transcriptomic profiles of cherry tomato fruit, harvested during postharvest storage under different temperatures and after 1-MCP treatment	(Zou et al., 2018)
tomato	multi-omics	appearance, flavor, texture	different tomato genotypes follow distinct transcriptomic, metabolomic	(D’Esposito et al., 2017)
tomato	multi-omics	flavor, size, and production	RNA-seq were capture genes associated structural variants	(Alonge et al., 2020)
tomato	multi-omics	steroidal glycoalkaloids and pericarp metabolites	genes with major effect on fruit metabolome	(Zhu et al., 2018)
tomato	multi-omics	fruit flavor	identified new genes and a rare allele for fruit flavor	(Gao et al., 2019)
tomato	multi-omics	carotenoids	identified DEGs related to ripening and carotenoid pathways	(Zuo et al., 2020)
tomato	multi-omics	fruit quality (nitrate, soluble sugar, etc.)	identification of DEGs in tomato fruits under substrate-Based and soil cultivation conditions	(Guo et al., 2022)

### 3.1 Regulation of ascorbic acid metabolism

Ascorbic acid (AsA) is the most abundant antioxidant in plant cells. Pepper is among the vegetables with the highest AsA content (240 mg/100 g fresh weight) (Marín et al., 2004). In pepper, RNA-seq and targeted expression profiling have illuminated the molecular basis of nutritional quality and flavor compounds that define consumer preferences and commercial value. The expression levels of genes involved in AsA biosynthesis, recycling, and degradation have been analyzed in sweet pepper using quantitative real-time PCR, where an inverse correlation between AsA concentrations and the expression of the biosynthetic genes was observed (Alós et al., 2013).

### 3.2 Capsaicinoid biosynthesis and pungency determination

Capsaicinoids confer the characteristic pungency of chili peppers while providing protection against herbivores, insects, and microorganisms (Tewksbury and Nabhan, 2001). RNA-seq transcriptome profiling of pungent chili pepper ‘Xiaomila’ (C. frutescens) identified three structural genes encoding key enzymes in capsaicinoid biosynthesis: dihydroxy acid dehydratase (DHAD), threonine deaminase (TD), and prephenate aminotransferase (PAT) (Liu et al., 2013). Developmental time-course analysis in Serrano ‘Tampiqueño’ chili pepper revealed distinct temporal dynamics for these biosynthetic genes: DHAD expression remained stable during early fruit development (10–40 days post-anthesis, DPA) before declining at maturity (40–60 DPA), while TD expression decreased progressively throughout all developmental stages (Martínez-López et al., 2014). Comparative transcriptomics between pungent and non-pungent pepper placental tissue across development, alongside orthologous gene expression in tomato, identified the evolutionary innovations underlying capsaicinoid biosynthesis (Kim et al., 2014). This study revealed that activation of branched-chain amino acid aminotransferase ketoacyl-ACP synthase, and the acyltransferasegene during evolution within Solanaceae enabled the specialized biosynthetic capacity of chili peppers. These genes showed elevated expression specifically in pungent fruit placental tissue, confirming the tissue-specific nature of capsaicinoid biosynthesis and providing candidate markers for pungency breeding programs.

### 3.3 Ethylene-regulated ripening and flavor compound biosynthesis

Tomato has served as the archetypal model for understanding climacteric fruit ripening, where ethylene orchestrates dramatic metabolic transitions affecting color, flavor, and texture (Cara and Giovannoni, 2008; Mathieu et al., 2009). RNA-seq analysis of fruits treated with 1-methylcyclopropene (1-MCP), an ethylene inhibitor that extends shelf-life, identified 5,683 significantly differentially expressed genes (DEGs), revealing the extensive transcriptional network under ethylene control (Bobokalonov et al., 2018). 1-MCP treatment downregulated genes governing ethylene synthesis and signal transduction alongside carotenoid biosynthetic genes, linking ethylene perception to pigment accumulation that defines tomato fruit color. Integration of RNA-seq with DNA methylation profiling further demonstrated that ripening-associated genes, including ethylene biosynthesis enzymes (ACO2), signaling components (CTR1), ethylene response factors (ERF2, ERF5, ERF17, ERF114), and carotenoid pathway genes (PSY1, NSY, CrtR-b2, LCY1, NCED), are regulated through coordinated transcriptional and epigenetic mechanisms (Zuo et al., 2020).Storage temperature interactions with ethylene signaling profoundly affect flavor volatile production. Comparative transcriptomics across storage temperatures (25°C, 10°C, and 4°C) revealed that cold storage (4°C) suppressed expression of five genes responsible for biosynthesis of terpenoids, alcohols, and ester; key contributors to tomato flavor compared to moderate refrigeration (10°C) (Zou et al., 2018). These findings provide molecular explanations for the well-known consumer complaint that refrigerated tomatoes lack flavor, demonstrating that transcriptional suppression of volatile biosynthesis rather than metabolite degradation underlies this quality loss.

### 3.4 Phenylalanine and fatty acid-derived flavor networks

Beyond ethylene-regulated processes, tomato flavor depends heavily on phenylpropanoid and fatty acid-derived volatiles. Phenylalanine serves as a precursor for numerous flavor compounds, including methyl salicylate, a key phenylpropanoid contributor to tomato aroma (Tieman et al., 2010; Dal Cin et al., 2011; Kaur et al., 2023). Paradoxically, RNA-seq analysis revealed that organic nutrient supplementation increased phenylalanine accumulation but decreased phenylalanine-derived volatiles, including methyl salicylate, due to altered expression of LeAADC, LePAR, and SlSAMT genes (Li et al., 2022b). This counterintuitive result demonstrates that precursor availability alone does not determine volatile production and transcriptional control of biosynthetic and regulatory genes governs metabolic flux, with implications for strategies aimed at improving flavor. Enzymatic browning in fresh-cut potato products, caused by polyphenol oxidase activity on phenolic substrates, represents another critical quality defect (Cabezas-Serrano et al., 2009). Comparative transcriptomics between browning-resistant (‘Kexin 13’) and browning-susceptible (‘Yunshu 505’) cultivars revealed that DEGs were enriched in lipid metabolism and amino acid metabolism pathways, with phenolic substrates produced through phenylpropanoid biosynthesis closely associated with browning development (Qiao et al., 2022; Darshan et al., 2020). These findings provide both candidate genes for marker development and physiological targets for breeding low-browning potato varieties suitable for fresh-cut processing markets.

Fatty acid-derived volatiles, encompassing both long-chain and short-chain compounds, constitute another major flavor class. Comparative transcriptomics between processing tomato M82 and an introgression line identified SlLIP1, encoding a lipase responsible for lipid degradation to fatty acids; SlLIP1 knockdown lines showed reduced long-chain fatty acid-derived volatiles (Garbowicz et al., 2018). Similarly, RNA-seq combined with volatile profiling identified SlLIP8 as governing short-chain fatty acid-derived volatile production; expression levels correlated strongly with Z-3-hexen-1-ol and hexyl alcohol content, and SlLIP8 mutants exhibited substantially reduced volatile accumulation (Li et al., 2020). These lipase genes represent promising targets for metabolic engineering or marker-assisted selection to enhance specific volatile profiles, enabling breeding for improved flavor intensity or novel aroma characteristics.

### 3.5 Anthocyanin and chlorogenic acid regulation in eggplant

In eggplant, anthocyanins and chlorogenic acid (CGA) are the predominant secondary metabolites, contributing to fruit pigmentation, antioxidant capacity, and nutritional value (Jiang et al., 2016). The MYB transcription factor *SmMyb1* emerged as a master regulator controlling both anthocyanin and CGA biosynthesis across eggplant tissues (Docimo et al., 2016). Quantitative PCR profiling revealed that SmMyb1 expression was relatively low across most tissues except stems, while functional analysis through transient overexpression in *N. benthamiana* demonstrated that C-terminal domain deletions specifically disrupted anthocyanin biosynthesis and CGA accumulation, indicating this region confers regulatory specificity. Furthermore, time-course RNA-seq analysis following light exposure (0, 0.5, 4, and 8 hours after bag removal) identified 1,956 DEGs, including 24 structural genes in anthocyanin biosynthesis pathways and 102 transcription factors exhibiting dynamic light-responsive expression changes (Li et al., 2018a). Notably, three photoreceptors: UV Resistance Locus 8 (*UVR8*), Cryptochrome 3 (*CRY3*), and (*UVR3)* were differentially expressed, along with light signal transduction elements COP1 and two SPA proteins, revealing the molecular cascade linking light perception to anthocyanin biosynthesis. This regulatory network provides targets for manipulating pigmentation patterns in eggplant breeding, with potential applications for developing cultivars with enhanced anthocyanin content for premium markets or specialized colors for consumer appeal.

Across Solanaceae crops, transcriptomic studies reveal several unifying principles governing secondary metabolite accumulation and quality trait expression. First, major regulatory hubs, including ethylene signaling in tomato, MYB transcription factors in eggplant, and temperature-responsive networks in potato coordinate expression of multiple biosynthetic pathways simultaneously, enabling pleiotropic effects from single genetic interventions. Second, environmental factors (temperature, light, nutrient availability) exert profound transcriptional effects that can override or modulate developmental programs, necessitating genotype-by-environment considerations in breeding. Third, counterintuitive relationships between precursor availability and end-product accumulation (exemplified by phenylalanine and AsA studies) highlight that transcriptional regulation of metabolic flux, rather than substrate concentration, often limits metabolite production. Finally, tissue-specific and temporal expression patterns indicate that effective breeding strategies must consider not just which genes to manipulate, but when and where they are expressed during development and post-harvest handling. These insights collectively enable more sophisticated breeding approaches that leverage expression-based markers, target regulatory genes for greater pleiotropic benefits, and account for environmental effects on quality trait expression.

## 4 Transcriptomics of solanaceous crops for biotic stress responses

Biotic stresses, including fungi, bacteria, viruses, nematodes, and insect pests represent the primary constraints limiting solanaceous crop productivity, with plant diseases alone causing substantial global yield losses (Kaur et al., 2019). While disease and pest management strategies include pathogen-free seed, chemical control, and cultural practices, deployment of resistant cultivars remains the most economically effective and environmentally sustainable approach (Mahmoud, 2021). However, resistance breeding requires precise identification and introgression of resistance genes (R-genes) alongside understanding of defense activation mechanisms (Lucas, 2011; Li et al., 2022a). Transcriptomic profiling, initially through microarrays and increasingly through RNA-seq has revolutionized this process by enabling genome-wide characterization of defense responses, identification of novel R-genes, and elucidation of regulatory networks governing pathogen recognition and downstream immunity.

### 4.1 Fungal pathogen defense mechanisms

Fungal pathogens constitute the predominant biotic threat to solanaceous crops, with late blight disease caused by *Phytophthora infestans* representing the most devastating example, inflicting up to 70% yield losses in both tomato and potato (Singh et al., 2021a). Comparative transcriptome analysis between *P. infestans*-infected resistant and susceptible tomato cultivars identified 1,037 DEGs and 688 long non-coding RNAs (lncRNAs), revealing previously unrecognized regulatory layers in disease resistance (Cui et al., 2017). Notably, lncRNA16397 was identified as an antisense transcript regulating expression of SlGRX22, a glutaredoxin gene involved in redox homeostasis; overexpression of both lncRNA16397 and SlGRX22 enhanced *P. infestans* resistance, demonstrating that non-coding RNAs represent untapped genetic resources for resistance breeding beyond traditional protein-coding R-genes.

The tomato-*Cladosporium fulvum* pathosystem, a classical model for studying gene-for-gene resistance interactions, exemplifies how transcriptomics elucidates R-gene function. RNA-seq analysis of *C. fulvum*-infected tomato cultivars carrying the Cf-19 resistance gene identified 418 DEGs mediating resistance responses, providing a comprehensive catalog of downstream genes activated by R-gene recognition (Zhao et al., 2019). This systems-level view reveals that single R-genes trigger coordinated expression changes across multiple defense pathways, informing expectations for resistance durability and potential trade-offs with growth or yield. In potato, transcriptomic studies have dissected defense responses to multiple necrotrophic and hemi biotrophic fungal pathogens. RNA-seq profiling of *Alternaria solani* (early blight) and *Rhizoctonia solani* (black scurf) infections revealed upregulation of biotic stress tolerance genes and pathogen defense transcripts, illuminating the distinct transcriptional signatures associated with necrotrophic pathogen lifestyles (Zrenner et al., 2020; Brouwer et al., 2021). Time-course transcriptomics of potato-*Fusarium sulphureum* (dry rot) interactions identified 1,025, 1,334, and 1,394 upregulated DEGs at 1-, 3-, and 7-days post-inoculation (dpi), respectively, with lignin biosynthesis genes, including *PAL*, *4CL*, *CAD*, and *POD* showing progressively elevated expression (Fan et al., 2021). This temporal profiling demonstrates that structural defense responses intensify over time, suggesting that early resistance assessment may underestimate cultivar differences and that breeding programs should evaluate defense responses across multiple infection stages.

Eggplant transcriptomics has leveraged wild relatives to identify resistance mechanisms absent in cultivated germplasm. While *Solanum melongena* is highly susceptible to the soil-borne fungus *Verticillium dahliae* (Verticillium wilt), the wild relative *S. aculeatissimum* exhibits robust resistance. Transcriptome analysis of *V. dahliae*-infected *S. aculeatissimum* roots revealed 11,696 upregulated and 5,949 downregulated genes, with enrichment in R-genes, pathogenesis-related (PR) proteins, and phenylpropanoid pathway genes (Zhou et al., 2016). This massive transcriptional reprogramming encompassing nearly 30% of the expressed genome indicates that resistance involves coordinated activation of multiple defense layers rather than single-gene effects, providing numerous targets for introgression into cultivated eggplant through marker-assisted breeding or genomic selection approaches.

### 4.2 Nematode resistance networks

Nematodes, particularly root-knot nematodes (RKNs) and cyst nematodes, inflict severe damage on vegetable crops through root system colonization. In potato, comparative transcriptomics between potato cyst nematode (PCN)-resistant (‘Kufri Swarna’) and susceptible (‘Kufri Jyoti’) cultivars identified 438 upregulated and 353 downregulated genes in the resistant line, with upregulated genes enriched for disease resistance genes and transcription factors including *WRKY, HMG*, and *MYB* families (Chandrasekar et al., 2022). These transcription factors likely serve as master regulators for coordinating expression of downstream defense genes, representing high-value targets for breeding interventions that could confer broad-spectrum resistance. Tobacco transcriptome profiling following Meloidogyne incognita (RKN) invasion revealed 4,354 differentially expressed genes in resistant genotypes, encompassing genes involved in cell wall modification, oxidative burst, signal transduction, and transcriptional regulation by *ERF, MYB*, and *WRKY* factors (Li et al., 2018c). The prominence of cell wall modification genes suggests that physical barriers preventing nematode penetration or migration constitute a key resistance mechanism. Similarly, transcriptome analysis of S. torvum, a wild eggplant relative used as nematode-resistant rootstock identified 5,360 DEGs following RKN infection, enriched in protein phosphorylation, defense signal transduction, and plant-pathogen interaction pathways (Zhang et al., 2021b). The convergence on signaling and phosphorylation pathways across multiple nematode-host systems indicates that rapid signal perception and amplification are critical for effective nematode resistance, suggesting that breeding for enhanced early-response signaling components could improve resistance across diverse nematode species.

### 4.3 Viral disease resistance mechanisms

Unlike fungal and bacterial diseases, viral infections in solanaceous crops lack effective chemical control options, rendering genetic resistance the sole viable management strategy. In pepper, chili leaf curl virus (ChiLCV) threatens production across South Asia. Suppression subtractive hybridization (SSH) followed by transcriptomic profiling of ChiLCV-infected resistant cultivar ‘Punjab Lal’ identified 231 unique ESTs related to cellular and physiological processes governing resistance (Kushwaha et al., 2015). Subsequent microarray analysis comparing susceptible and resistant Capsicum species identified 319 DEGs, with 234 unique genes upregulated more than 2-fold in resistant lines, revealing a broader resistance network than initially captured by SSH (Rai et al., 2016). The progression from SSH to microarray demonstrates how technological advances enable increasingly comprehensive characterization of resistance mechanisms.

In tomato, comprehensive transcriptomic analysis of tomato spotted wilt virus (TSWV)-infected plants carrying the Sw-7 resistance gene identified 1,244 DEGs involved in callose deposition, lignin accumulation, RNA silencing, and transcriptional regulation (Padmanabhan et al., 2019). Functional characterization revealed that PR-5 (pathogenesis-related protein 5) directly participates in Sw-7-mediated resistance, providing a specific molecular target for diagnostic marker development or engineering enhanced TSWV resistance. This study exemplifies how transcriptomics bridges the gap between R-gene identification and mechanistic understanding of resistance execution.

Tobacco transcriptomics has illuminated complex virus-betasatellite interactions. RNA-seq comparison between tobacco plants infected with Tobacco curly shoot virus (TbCSV) alone versus co-infection with Tobacco curly shoot betasatellite (TbCSB) revealed 3,196 and 4,081 DEGs, respectively, with 7 and 13 genes showing remarkable effects on brassinosteroid (BR) and jasmonic acid (JA) biosynthesis and signaling (Li et al., 2018b). The symptom shift from upward to downward leaf curling with betasatellite co-infection correlates with distinct transcriptional reprogramming of hormone pathways, demonstrating that virus-satellite interactions reprogram host physiology beyond the effects of either agent alone. These findings have implications for understanding disease complexes in solanaceous crops where multiple pathogens co-occur.

### 4.4 Insect pest defense and interspecific variation

Comparative transcriptomics between tomato and eggplant infested by leaf miner (*Tuta absoluta)* revealed higher expression levels of plant immunity-related transcripts in eggplant, suggesting differential constitutive or induced defense capacity between these solanaceous species (Chen et al., 2021). This interspecific variation indicates that genes underlying eggplant’s enhanced immunity could be introgressed into tomato through wide crosses or identified as targets for engineering improved insect resistance. Understanding species-specific defense architectures enables strategic exploitation of genetic diversity within Solanaceae for pest resistance breeding.

Despite pathogen diversity spanning fungi, nematodes, viruses, and insects, transcriptomic studies across solanaceous crops reveal convergent defense mechanisms and regulatory architectures. First, transcription factor families particularly WRKY, MYB, and ERF emerge repeatedly as master regulators coordinating pathogen-specific responses, representing high-leverage targets for breeding interventions that could provide broad-spectrum resistance. Second, phenylpropanoid pathway activation constitutes a universal defense response across pathogen types, producing antimicrobial compounds, lignin for physical barriers, and signaling molecules for systemic resistance. Third, pathogenesis-related (PR) proteins, including PR-5, chitinases, and β-1,3-glucanases, are consistently upregulated across resistance responses, suggesting that their expression levels could serve as quantitative predictors of resistance capacity. Furthermore, the discovery of lncRNAs regulating defense genes (lncRNA16397/SlGRX22) reveals previously unrecognized regulatory layers, expanding the pool of genetic variation accessible for resistance improvement beyond traditional R-gene pyramiding.

The massive transcriptional reprogramming observed in resistant responses up to 30% of the transcriptome in V. dahliae-resistant eggplant suggests that effective resistance often involves coordinated multi-gene networks rather than single-gene effects. This systems-level perspective supports genomic selection approaches that capture small-effect loci collectively contributing to quantitative resistance, complementing traditional R-gene deployment. Finally, wild relatives (*S. aculeatissimum, S. torvum*) harbor resistance mechanisms absent in cultivated germplasm (Zhang et al., 2021b), and transcriptomics provides the molecular roadmap for identifying and introgressing these valuable alleles into elite breeding lines, accelerating the development of durable, broad-spectrum resistance across solanaceous crops (**Table 2**).

**Table 2:** Description of several transcriptomics techniques used to study biotic stresses in solanaceous crops.

Crop	Technique	Biotic stress (organism)	Trait	References
tomato	RNA-seq	Tomato yellow leaf curl virus (TYLCV)	DEGs involved in cell wall reorganization, transcriptional regulation, defense response, ubiquitination, metabolite synthesis	(Chen et al., 2013)
tomato	microarray	*Botrytis cinerea*	profile the expression of genes for the biosynthesis, modification and signal transduction	(Blanco-Ulate et al., 2013)
tomato	RNA-seq	*C. globosum*	deciphered the defense signaling pathways associated with systemic resistance induced by *Chaetomium globosum*	(Singh et al., 2022)
tomato	RNA-seq	tomato spotted wilt virus (TSWV), cucumber mosaic virus (CMV), and potato virus Y (PVY)	grafting alters tomato transcriptome that increases tolerance to an airborne virus infection	(Spanò et al., 2020)
tomato	RNA-seq	*Verticillium dahliae*	molecular mechanisms of tomato plant susceptibility	(Tan et al., 2015)
tomato	microarray	*Ralstonia. solanacearum*	elucidate the molecular mechanisms in the early resistance response	(Ishihara et al., 2012)
tomato	microarray	*Ralstoniasolanacearum*	transcript level of biochemical and immunohistochemical work on silicon induced resistance	(Ghareeb et al., 2011)
tomato	RNA-seq	TSWV	DEGs were identified throughout a disease progression process involving networks of host resistance genes, RNA silencing/antiviral defense genes, and crucial transcriptional and translational regulators.	(Padmanabhan et al., 2019)
tomato	RNA-seq	*Phytophthora infestans*	identification of lncRNA16397 conferring resistance by co-expressing glutaredoxin	(Cui et al., 2017)
tomato	RNA-seq	*Phytophthora infestans*	SpWRKY3 was screened from transcriptome analysis of tomatoes inoculated with and without pathogens	(Cui et al., 2018)
tomato	RNA-seq	*Cladosporium fulvum*	analyse the differences between the response of resistant plants (carrying the Cf-19 gene) and susceptible plants (Moneymaker (MM), carrying the Cf-0 gene) at 0, 7 and 20 days after inoculation	(Zhao et al., 2019)
tomato	RNA-seq	*Clavibacter michiganensis* subsp. *michiganensis*	transcriptome response of resistant and susceptible tomato lines the infection	(Basim et al., 2021)
tomato	RNA-seq	*Meloidogyne incognita*	resistance against root-knot nematode	(Shukla et al., 2018)
tomato	RNA-seq	TYLCV	determine the distribution of the viral transcripts accumulated during the infection	(Piedra-Aguilera et al., 2019)
tomato	microarray/RNA-seq	Potato spindle tuber viroid	transcriptomic profile changes in tomato roots systemically infected with mild or severe PSTVd variants	(Góra-Sochacka et al., 2019)
potato	RNA-seq	Potato Virus A (PVA)	identify the genes involved in the response to PVA infection and potential candidate genes for improving resistance cultivars	(Li et al., 2017)
potato	RNA-seq	*Phytophthora infestans (Pi)*	exogenous ethylene activates immune and defense responses in potato against late blight	(Yang et al., 2020a)
potato	RNA-seq	*P*. *infestans*	genes associated with late blight resistance in potato	(He et al., 2021)
potato	microarray	PVY	transcript and protein abundance of potato following the infection with PVY	(Stare et al., 2017)
potato	RNA-seq	*Alternaria solani*	identified the differential expression of several potato and *A. solani* transcripts that present a group of valuable candidates into the roles in immunity or disease development.	(Brouwer et al., 2021)
potato	RNA-seq	*Ralstonia solani*	necrotrophic lifestyle of *R. solani* AG3-PT during early interaction with its host	(Zrenner et al., 2020)
potato	RNA-seq	*Fusarium sulphureum.*	involvement of lignin synthesis-related pathway and MAPK signaling pathway-plant in resistance	(Fan et al., 2021)
potato	RNA-seq	*Synchytrium endobioticum*	transcriptome analysis of resistance mechanism to potato wart disease	(Li et al., 2021a)
potato	RNA-seq	Potato cyst nematodes	identification of disease resistance genes and molecular markers for PCN infestation	(Chandrasekar et al., 2022)
potato	DeepSAGE	*P. infestans*	DeepSAGE transcriptome analysis uncovered novel candidate genes for plant host pathogen interactions	(Gyetvai et al., 2012)
pepper	RNA-seq	*Xanthomonas campestris* pv. *vesicatoria*	DEGs from transcriptomic analysis of a susceptible cultivar and a resistant cultivar were identified	(Gao et al., 2021)
pepper	RNA-seq	*Colletotrichum capsici*	resistance mechanism and identification of markers for anthracnose resistance in chili	(Kumar et al., 2022)
pepper	RNA-seq	Chilli leaf curl virus(Chi LCV)	protein homeostasis and defense in resistant chili plants against Chi LCV infection	(Kushwaha et al., 2015)
eggplant	RNA-seq	*V. dahliae*	transcriptome of the wild eggplant species *S. aculeatissimum* in response to *V. dahliae*	(Zhou et al., 2016)
eggplant	RNA-seq	*Tuta absoluta*	transcriptome analysis showed higher transcript changes in infested eggplant than tomato	(Chen et al., 2021)
eggplant	RNA-seq	*Meloidogyne incognita* (root-knot nematode - RKN)	transcriptomic profiles of eggplant and Solanum torvum were compared. Response-related DEGs were identified as being involved in stimulus response, protein phosphorylation, hormone signaling, and plant-pathogen interactions.	(Zhang et al., 2021b)
tobacco	RNA-seq	Tobacco curly shoot virus (TbCSV) and Tobacco curly shoot betasatellite (TbCSB)	transcriptome analysis *N. benthamiana* infected by TbCSV	(Li et al., 2018b)
tobacco	RNA-seq	*Cucumber mosaic virus* (CMV)	mechanisms of CMV tolerance in plants	(Liu et al., 2019)
tobacco	RNA-seq	CMV	analysis of *N. tabacum* infected by CMV during systemic symptom development	(Lu et al., 2012)
tobacco	RNA-seq	*Phytophthora nicotianae*	candidate resistant and susceptible genes against *P. nicotianae* infections	(Meng et al., 2021)
tobacco	RNA-seq	*M. incognita*	defense-related genes against *M. incognita* invasion in tobacco	(Li et al., 2018c)

## 5 Transcriptomics for solanaceous crops for abiotic stress responses

Plants in natural settings are confronted by multiple abiotic stresses including drought, salinity, temperature extremes, and nutrient limitations that cause significant crop yield losses (Kaur et al., 2022). These stresses ultimately generate reactive oxygen species (ROS) that interfere with protein-protein interactions, disrupt ionic and osmotic homeostasis, and cause tissue and cell death (Singh et al., 2021b), severely hampering physiological growth, yield, and productivity. In response, plants have evolved adaptive mechanisms operating at molecular, cellular, and physiological levels, with transcriptional reprogramming representing the first line of defense (Gong et al., 2020). Central to these responses are transcription factors (TFs); trans-acting regulatory proteins that bind promoter regions to control expression of target genes governing stress tolerance. To date, sixty TF families have been identified in solanaceous crops, with WRKY, DOF, MYB, bZIP, ARF, ERF, HSF, and NAC families most prominently implicated in abiotic stress defense (Tolosa and Zhang, 2020). Transcriptome profiling across tissue types and developmental stages has enabled systematic identification of functional genes and regulatory networks underlying stress tolerance, providing the molecular foundation for breeding climate-resilient cultivars capable of maintaining productivity under increasingly variable environmental conditions.

### 5.1 Drought and salinity stress responses in tomato

Tomato, sensitive to heat, salinity, cold, and drought stress, has served as the primary model for dissecting abiotic stress transcriptional networks in Solanaceae. Comparative transcriptomics under drought and salinity stress identified the bZIP transcription factors *SlAREB1* and *SlAREB2* as master regulators, with expression induced in both leaves and root tissues (Orellana et al., 2010). Microarray and cDNA-amplified fragment length polymorphism (cDNA-AFLP) analysis of *SlAREB1*-overexpressing lines revealed production of oxidative stress-response proteins, defense-related proteins, protease inhibitors, and additional transcription factors, demonstrating that single TF manipulations can trigger cascading activation of entire stress tolerance networks. Key candidate genes associated with drought tolerance included *AHG2* (ABA signaling), *PRXIIF* and *PRXQ* (peroxidases for ROS detoxification), *SAP5* (stress-associated protein), and *CCD1* (carotenoid cleavage dioxygenase), collectively spanning signaling perception, ROS management, and metabolic adjustment.

For salinity tolerance specifically, critical candidate genes mapped to HKT1 (high-affinity potassium transporter) on chromosome 7 and LeNHX3 (Na+/H+ exchanger) on chromosome 1, which regulate ion homeostasis by controlling sodium exclusion and compartmentalization. RNA-seq analysis of tomatoes growing under seawater salinity stress showed upregulation of DEGs responsible for growth maintenance, signal transduction, and protein-binding pathways (Usharani et al., 2022), indicating that salinity tolerance requires coordinated transcriptional activation spanning multiple cellular processes beyond ion transport alone. Functional analysis under combined drought and salinity stress revealed elevated levels of hydrogen peroxide (signaling molecule), melatonin (antioxidant), and zeatin riboside (cytokinin) during stress conditions (Zhou et al., 2019) (**Table 3**), demonstrating that plants deploy overlapping regulatory mechanisms for multiple concurrent stresses; a critical consideration for breeding cultivars adapted to real-world field conditions where multiple stresses co-occur.

**Table 3:** Description of different TFs and regulation of genes during abiotic stress conditions in plants.

Crop	Technique	Abiotic stress	Characteristics	References
eggplant	RNA-seq	low temperature	plant hormone signal transduction related genes were involved in eggplant response to low temperature	(Yang et al., 2020b)
tomato	RNA-seq	drought	In-depth analysis of DEGs revealed that ABA metabolism and polyamine biosynthesis elevated to provide tolerance	(Nie et al., 2022)
potato	RNA-seq	heat	genes involved in amino acid biosynthesis and secondary metabolism were mostly induced during heat exposure	(Liu et al., 2021a)
potato	RNA-seq	heat	DEGs were clustered into 49 different GO types, reflecting the functional diversity of the heat stress response genes	(Tang et al., 2020)
potato	RNA-seq	cold	the expression of 15 heat shock proteins and the accumulation of soluble sugars (mediated by enzymes such as invertase inhibitor and fructokinase) to prevent cellular damage	(Lin et al., 2019)
potato	RNA-seq	drought	differential gene expression trend reflected the upregulation of genes involved in carbohydrate metabolism, flavonoid biosynthesis, lipid biosynthesis/transfer, heat shock proteins and secondary metabolites like phenolics and lignins.	(Aliche et al., 2021)
potato	RNA-seq	nitrogen deficiency	DEGs like glutamate dehydrogenase, glutamine synthetase and carbonic anhydrasehelped to combat nitrogen stress	(Zhang et al., 2020)
pepper	RNA-seq	heat, cold, salinity, and osmotic stress	Analyses of the transcriptome data in this study will provide useful information for basic studies of various stimuli to facilitate the development of stress-resistant pepper cultivars	(Kang et al., 2020)
pepper	RNA-seq	heat	DEGs revealed the most enriched pathways were metabolic processes under stress and photosynthesis and light harvesting during stress	(Wang et al., 2021)
pepper	microarray	salt	stress signaling genes played important role in heat stress	(López−Serrano et al., 2021)
pepper	RNA-seq	drought	calcineurin B-like proteins and CBL-interacting protein kinases are important TFs during multiple stress conditions	(Ma et al., 2021b)
tobacco	RNA-seq	chilling stress/low temperature	DEGs were primarily enriched in the following functions: regulating cellular osmotic balance, promoting photosystem II repair, modulating signal transduction pathways such as ABA/GA, and enhancing lipid metabolism and lignin synthesis to improve membrane stability and cell wall mechanical strength	(Zhou et al., 2020)
tobacco	RNA-seq	salt stress	the up-regulated genes in salt stress were mainly concentrated in transcription factor WRKY family and PAR1 resistance gene family	(Wu et al., 2021)
tobacco	RNA-seq	drought	upregulation of genes involved in arginine, proline, and starch metabolism	(Khan et al., 2019)
tobacco	RNA-seq	salinity	acetylcholine plays an important role in downregulating ROS and hydrogen peroxide level	(Qin et al., 2021)
tobacco	microarray	environmental changes	DEGs among three cultivated regions were associated with the light reaction of photosystem II, response to stimuli, and secondary metabolism.	(Lei et al., 2013)

### 5.2 Heat shock response and temperature stress adaptation

Heat stress transcriptional responses have been extensively characterized across multiple solanaceous crops, revealing conserved heat shock factor (HSF) regulatory networks. In tomato, RNA-seq analysis showed that spermidine, a polyamine plays an important regulatory role in heat stress tolerance, while endoplasmic reticulum (ER) stress responses constitute a critical factor contributing to varietal heat tolerance differences (Usharani et al., 2022). The connection between ER stress and heat tolerance suggests that protein folding capacity and quality control mechanisms limit thermotolerance, providing targets for breeding interventions.

In potato, candidate genes encoding heat shock transcription factors including *StHsf004, StHsf007, StHsf009, StHsf014, and StHsf019* showed constitutive expression under non-stress conditions, providing basal thermotolerance capacity, while additional HSFs were induced specifically under heat stress (Tang et al., 2016). Comprehensive transcriptome analysis of heat-sensitive potato variety ‘Agria’ identified 2,949 differentially expressed genes responding to heat stress, resulting in accumulation of heat shock proteins that serve as molecular chaperones preventing protein denaturation and aggregation (Usharani et al., 2022). In pepper, RNA-seq comparison between heat-tolerant and heat-sensitive varieties revealed that the glutathione metabolic pathway plays a critical role in heat tolerance (Wang et al., 2020), providing antioxidant capacity to neutralize heat-induced ROS production. These findings collectively indicate that heat tolerance involves both protective protein expression (chaperones) and ROS management systems, suggesting that breeding strategies should target both mechanisms simultaneously.

Combined drought and heat stress transcriptomics in potato revealed differential regulation of at least 19,000 transcripts, including genes encoding type-1 metacaspase (DMT400032693, programmed cell death), ABI5-binding protein 2 (DMT400036544, ABA signaling), cell wall invertase (DMT400023092, sugar metabolism), tryptophan synthase (DMT400029363, amino acid biosynthesis), and lipid biosynthesis enzymes (DMT400033492) (Demirel et al., 2020). Critically, tolerant varieties encoded fewer transcripts involved in programmed cell death pathways, suggesting that stress tolerance involves not just activating protective mechanisms but also suppressing cell death signaling, a balance that determines whether plants survive or succumb to stress combinations.

### 5.3 Cold stress and low-temperature acclimation

Cold stress responses involve distinct transcriptional programs from heat stress, with phenylpropanoid pathway activation emerging as a common defense strategy. In potato, functional analysis identified microRNAs stu-novel-miR28211 and stu-novel-miR43095 as regulators of cold tolerance, modulating peroxidase levels in the phenylpropanoid pathway (Liao et al., 2021). The phenylpropanoid pathway produces defensive metabolites, lignin for cellular reinforcement, and antioxidant compounds representing a multi-layered cold protection strategy.

In eggplant, cold stress induced expression of WRKY transcription factors SmWRKY26 and SmWRKY32 (Yang et al., 2020b), while paradoxically downregulating anthocyanin biosynthesis genes, including CHI, 3GT, F3′5′H, DFR2, ANS, and associated transcription factors resulting in lower anthocyanin content at high temperatures (Lv et al., 2019). This temperature-dependent metabolic shift suggests that anthocyanin biosynthesis is optimized for moderate temperatures, with resources redirected toward other protective mechanisms under temperature extremes.

Potato tuber quality during storage presents distinct challenges related to cold-induced sweetening and enzymatic browning. Combined transcriptomic and proteomic analysis of tubers stored at 15°C, 4°C, and 0°C revealed that cold stress triggered soluble sugar accumulation through coordinated regulation of granule-bound starch synthase 1, beta-amylase, invertase inhibitor, and fructokinase (Lin et al., 2019). This cold-induced sweetening reduces processing quality by promoting undesirable browning during frying, a major economic concern for the potato industry. Understanding the transcriptional basis of sugar accumulation enables breeding for cold-sweetening resistant cultivars or identification of storage conditions that minimize quality deterioration.

In tobacco, transcriptome analysis following chilling stress revealed upregulation of 1,170 genes and downregulation of 505 genes at 12- and 24-hours post-treatment (Zhou et al., 2020). These genes regulate cell osmotic potential and soluble sugar levels while promoting PSII (photosystem II) repair, lipid metabolism adjustments, and lignin synthesis to enhance cell wall mechanical strength. The emphasis on PSII repair highlights that photosynthetic apparatus damage represents a primary mechanism of cold injury, and tolerance requires active repair systems rather than passive protection alone.

### 5.4 Salinity tolerance mechanisms across species

Beyond tomato, salinity transcriptomics in other solanaceous crops has revealed both conserved and species-specific tolerance mechanisms. In salt-tolerant eggplant variety ‘ST118’, DEGs included transcription factors from C2C2-CO-like, WRKY, and MYB families, alongside ion transport genes AKT1 and KAT1 (potassium channels) and SOS1 (salt overly sensitive 1, Na+/H+ antiporter) (Li et al., 2018a). The co-regulation of transcription factors and ion transporters indicates that effective salinity tolerance requires both sensing/signaling components and effector mechanisms for ion homeostasis.

In tobacco, RNA-seq analysis identified 1,654 DEGs involved in salt stress responses, with upregulation of WRKY family and PAR1 family transcription factors providing salinity tolerance (Wu et al., 2021). This study also revealed the critical role of abscisic acid (ABA) in mediating salt resistance, with ABA-responsive genes constituting a major component of the salinity tolerance network. Transgenic tobacco seedlings overexpressing ArSQE (squalene epoxidase) along with bZIP transcription factors showed tolerance to multiple abiotic stresses (Upadhyay et al., 2020), demonstrating that manipulating key metabolic enzymes alongside regulatory factors can confer broad stress tolerance, a promising strategy for developing multi-stress resilient cultivars.

### 5.5 Drought tolerance networks and signaling cascade

Despite diversity in stress types and solanaceous species, transcriptomic studies reveal several convergent molecular principles governing abiotic stress tolerance. First, a core set of transcription factor families particularly WRKY, MYB, bZIP, ERF, and NAC function as master regulators across all stress types and species, indicating that these TF families possess inherent stress-responsive regulatory capacity. Manipulating expression or DNA-binding specificity of these master regulators represents a high-leverage breeding strategy potentially conferring tolerance to multiple stresses simultaneously.

Second, ROS management through antioxidant systems (glutathione metabolism, peroxidases, carotenoid-derived signals) constitutes a universal stress response mechanism, as ROS generation is the common downstream consequence of most abiotic stresses. Breeding for enhanced antioxidant capacity through selection on glutathione pathway genes or peroxidase expression levels could provide baseline stress tolerance across diverse environmental challenges.

Third, programmed cell death pathways emerge as critical determinants of stress outcomes: tolerant genotypes suppress cell death signaling while maintaining protective responses, whereas susceptible genotypes undergo premature tissue death. This suggests that stress tolerance breeding should focus not only on activating protective mechanisms but also on dampening cell death pathways, a dual strategy rarely considered in conventional breeding programs.

Fourth, the massive transcriptional reprogramming under combined stresses (19,000 transcripts in potato drought+heat stress) indicates that tolerance to stress combinations cannot be predicted from single-stress responses. This necessitates phenotyping and transcriptomic profiling under realistic multi-stress conditions that mirror field environments, rather than controlled single-stress treatments that may poorly predict field performance.

Fifth, tissue-specific and temporal dynamics of stress responses (e.g., SlAREB expression in both leaves and roots; time-course changes in tobacco chilling response) highlight that effective stress tolerance requires coordinated responses across organs and sustained transcriptional commitment over time. Breeding programs should therefore evaluate stress tolerance across multiple tissues and time points to capture genotypes with robust, sustained responses rather than transient stress reactions.

Finally, the discovery of regulatory microRNAs (stu-novel-miR28211, stu-novel-miR43095) and hormone signaling components (ABA, melatonin, cytokinins) reveals additional regulatory layers beyond protein-coding genes. Expanding breeding targets to include regulatory RNAs and hormone pathway components could unlock genetic variation inaccessible through traditional gene-focused approaches. These transcriptomic insights collectively provide a molecular roadmap for developing climate-resilient solanaceous cultivars capable of maintaining productivity under the increasingly variable and extreme environmental conditions projected for agricultural regions worldwide (**Table 3**).

## 6 Transcriptomic approaches to dissecting yield architecture and productivity

Yield represents the ultimate breeding target, yet its highly polygenic nature and sensitivity to environmental conditions have historically made it challenging to dissect genetically. Transcriptomics provides a powerful approach for understanding yield determination by capturing gene expression dynamics across tissues, developmental stages, and environmental conditions revealing regulatory networks that translate genetic variation into phenotypic outcomes (Lowe et al., 2017). Unlike single-timepoint genomic analyses, transcriptome profiling captures the active molecular processes governing resource allocation, organ development, and stress integration that collectively determine productivity. In solanaceous crops, yield improvement requires simultaneous optimization of fruit or tuber morphology, nutrient use efficiency, photosynthetic capacity, and reproductive development—all traits amenable to transcriptomic dissection. Moreover, as emphasized in the introduction, achieving genetic gain under climate change necessitates understanding how yield-determining processes respond to heat, drought, and other stresses. This section examines how transcriptomics has illuminated the molecular basis of yield components, providing actionable targets for breeding higher-yielding, climate-resilient cultivars.

### 6.1 Fruit and tuber morphology: developmental programs determining harvestable yield

Fruit size and shape constitute primary yield determinants in solanaceous crops, with morphological variation arising from early developmental programs that establish organ identity and growth trajectories. In tomato, genetic and transcriptomic analyses identified ENO (enolase) as a regulator of fruit size, with RNA-seq revealing that a promoter mutation selected during domestication increased ENO expression in cultivated tomatoes, driving larger fruit development (Yuste-Lisbona et al., 2020). This finding demonstrates that cis-regulatory variation, rather than protein-coding changes, can generate major morphological shifts, suggesting that breeding programs should screen regulatory regions, not just coding sequences, to unlock yield-enhancing alleles.

In potato, tuber shape determination involves complex developmental transitions from stolon to tuber. Combined genetic and transcriptomic analysis using RNA-seq across tissue sections identified five candidate genes governing tuber morphology: Soltu.DM.10G018510 (nuclear shuttle interacting protein), Soltu.DM.10G018550 (aminophospholipid ATPase, membrane trafficking), Soltu.DM.10G018580 (seven-transmembrane receptor), Soltu.DM.10G018620 (HSI2-like transcriptional repressor), and Soltu.DM.10G018660 (lipid transfer protein) (Fan et al., 2022). Critically, transcriptome sequencing revealed that developmental divergence between round and elongated tuber morphologies initiates as early as the stolon hook stage, much earlier than previously recognized. This temporal resolution demonstrates that yield-determining developmental decisions occur during vegetative rather than reproductive phases, indicating that breeding for tuber shape requires screening at early developmental stages and that selection based on mature tuber phenotypes may miss critical genetic variation affecting early developmental commitment.

In eggplant, comparative transcriptomics between four wild accessions (Solanum insanum) and 16 domesticated lines identified genomic regions targeted during domestication that could enhance yield improvement (Page and Chapman, 2021). Analysis of fixation index (F_ST_) and nucleotide diversity (π) revealed low diversity in domesticated populations, indicating selection sweeps in yield-related regions. These domestication-targeted regions likely harbor alleles balancing fruit size against plant vigor or stress tolerance, trade-offs that could be re-optimized through genomics-informed breeding that reintroduces favorable wild alleles into elite backgrounds.

### 6.2 Integrating morphology with stress tolerance: multi-trait transcriptional networks

An innovative study demonstrated that SlMX1, encoding a MIXTA-like MYB transcription factor that regulates trichome formation, simultaneously influences yield, stress tolerance, and secondary metabolism (Ewas et al., 2022). Comparative transcriptomics between SlMX1-overexpressing transgenic tomatoes and wild-type plants revealed that SlMX1 upregulated and downregulated genes governing cell growth, primary metabolism, and secondary metabolite biosynthesis, ultimately improving fruit yield while enhancing resistance to both biotic and abiotic stresses. This pleiotropic TF represents an ideal breeding target for addressing the consumer-producer trade-off. The identification of such master regulators through transcriptomics enables breeding strategies that avoid negative genetic correlations by selecting regulatory variants affecting multiple beneficial traits concurrently.

Similarly, an unexpected finding demonstrated that RNA N6-methyladenosine (m6A) modification, an epitranscriptomic layer regulating mRNA stability and translation increases potato yield and biomass in field trials (Yu et al., 2021). This reveals that yield improvement can be achieved not just through altering which genes are transcribed, but through modulating post-transcriptional RNA processing, expanding the pool of genetic variation accessible for breeding beyond traditional expression quantitative trait loci (eQTL).

### 6.3 Nutrient use efficiency: metabolic networks linking resource acquisition to productivity

Nitrogen use efficiency (NUE), the capacity to maintain productivity under limiting nitrogen availability represents a critical breeding target for sustainable agriculture and yield stability under variable nutrient conditions. In potato, RNA-seq analysis identified superoxide dismutase (ROS detoxification), probable protein phosphatase 2C (signaling), and high-affinity nitrate transporters as major candidate genes governing NUE. The involvement of ROS detoxification enzymes indicates that nitrogen limitation generates oxidative stress, and that NUE requires coordinated upregulation of both nitrogen acquisition and antioxidant systems, a systems-level understanding unattainable from genomic analysis alone.

In tobacco, RNA-seq following nitrogen deficiency identified 428 upregulated and 213 downregulated genes with distinct pathway enrichment patterns (Li et al., 2021b). Upregulated genes were enriched in MAPK signaling (stress perception), sesquiterpenoid and triterpenoid biosynthesis (secondary metabolism), and arginine and proline metabolism (compatible solutes), while downregulated genes were enriched in photosynthesis, nitrogen metabolism, and amino acid biosynthesis. This transcriptional reprogramming reveals an apparent paradox: nitrogen-deficient plants downregulate nitrogen assimilation and amino acid biosynthesis, the very processes needed to utilize available nitrogen. This suggests that severe nitrogen limitation triggers a resource conservation strategy prioritizing survival (stress signaling, compatible solutes) over growth, with implications for fertilization timing: applications must precede severe deficiency to maintain productive metabolism.

In eggplant, comparative transcriptomics between nitrogen-use efficient and inefficient genotypes revealed that genes involved in light reactions, particularly light-harvesting complexes (LHCs) and ferredoxin-NADP reductase (FNR) were upregulated in efficient genotypes (Mauceri et al., 2021). This counterintuitive finding indicates that NUE depends critically on photosynthetic efficiency: efficient genotypes maintain higher photosynthetic capacity under nitrogen limitation, generating sufficient carbon skeletons and ATP to maximize utilization of available nitrogen. This demonstrates that breeding for NUE requires selecting for maintained photosynthetic function under nutrient stress, not just enhanced nitrogen uptake, a physiological target that could be achieved through marker-assisted selection on LHC and FNR expression levels.

### 6.4 Inflorescence architecture and reproductive development

Inflorescence architecture directly determines potential fruit number per plant, representing a major yield component. Meristem transcriptomic profiling in tomato revealed that allelic dosage at specific loci controlling inflorescence branching improves architecture and yield (Soyk et al., 2017), demonstrating that yield increases can be achieved through optimizing allelic combinations rather than identifying novel genes, a principle applicable to genomic selection strategies that capture small-effect quantitative trait loci collectively contributing to complex traits.

Stigma position, which affects pollination efficiency and fertilization success, represents another critical reproductive trait. Comparative transcriptomics between exserted-stigma (improved pollination) and inserted-stigma genotypes identified 801 DEGs—566 upregulated, 235 downregulated (Riccini et al., 2021). Upregulated genes in exserted-stigma lines included those governing cell wall structure and metabolism, particularly sucrose metabolism genes (std1, sps, SuSy), the auxin efflux facilitator PIN-FORMED3, and two xyloglucan endotransglucosylase-hydrolases (XTH1, XTH7) mediating cell wall expansion. Hormone-related genes were prominently affected, including three Auxin Response Factors (ARFs), two Auxin/Indole-3-Acetic Acid proteins (Aux/IAA), two Arabidopsis Response Regulators (ARRs), and two GIBBERELLIN-STIMULATED TRANSCRIPT 1 (GAST1)-like genes. The convergence on auxin and gibberellin pathways indicates that stigma elongation results from coordinated hormone signaling driving localized cell expansion, providing candidate genes for breeding improved pollination efficiency under heat stress conditions that disrupt normal stigma development.

Parthenocarpy, fruit development without fertilization offers yield stability under pollination-limiting conditions (heat stress, pollinator scarcity). Microarray analysis of a parthenocarpic tomato mutant (pat) with pleiotropic floral traits identified novel genes regulating fruit set (Ruiu et al., 2015). Differentially expressed genes segregated into regulatory function (RF) and pollination-dependent (PD) groups. RF genes including LeT6/TKn2 (KNOTTED-like homeobox), SlBPEp, SlCRCb, and SlDEF showed downregulation in the pat mutant, while PD genes enriched in auxin perception and response including ARF and Aux/IAA family members (SlARF9, SlIAA14) exhibited altered expression patterns. This regulatory network dissection provides molecular handles for engineering facultative parthenocarpy that maintains normal fruit development under optimal conditions while enabling fruit set under stress, a breeding objective directly addressing climate resilience.

During fruit ripening, oligonucleotide microarray analysis revealed that most plastid genes are transcriptionally downregulated (Kahlau and Bock, 2008), with transcriptional, post-transcriptional, and translational controls all contributing to developmental regulation of plastid gene expression. This multilayered regulation of photosynthetic capacity during ripening affects carbon partitioning between growth and fruit filling, with implications for breeding optimized source-sink relationships.

### 6.5 Photosynthetic optimization and light response networks

Photosynthetic efficiency directly determines carbon fixation capacity and ultimately yield potential. Leaf morphology affects photosynthetic efficiency through altered light interception and physiological parameters, impacting fruit sugar accumulation and yield (Nicotra et al., 2011). RNA-seq analysis investigating associations between photosynthetic gene expression and leaf morphology in tomato identified transcriptional programs linking leaf shape variation to photosynthetic capacity (Chitwood et al., 2013), providing candidate genes for breeding leaf architectures optimized for specific canopy management or planting densities.

A dominant locus for continuous light tolerance on chromosome 7, identified through RNA-seq, increased yield by 20% in modern tomato hybrid lines carrying the introgressed allele (Velez-Ramirez et al., 2014). This substantial yield gain from manipulating photoperiod response demonstrates that breeding for altered circadian regulation or photoperiod sensitivity could unlock major productivity increases, particularly for protected cultivation systems where light duration can be controlled.

In pepper, RNA-seq analysis of CO_2_ enrichment responses identified 149 DEGs promoting photosynthesis and yield (Zhang et al., 2021a). Two genes, Capana00g004478 (downregulated) and Capana08g001515 (upregulated) both homologous to AtBGLU45 encoding β-glucosidase involved in carbohydrate and glutathione metabolism, showed opposite expression changes, suggesting antagonistic regulation of carbon partitioning and ROS management under elevated CO_2_. These findings have implications for breeding cultivars optimized for protected cultivation with CO_2_ supplementation or for adaptation to rising atmospheric CO_2_ levels.

Light quality also profoundly affects yield through altered photosynthetic gene expression. RNA-seq analysis of red/blue light treatments in pepper identified 96 genes involved in light reaction pathways, with expression of CPRF2, Paggis, HLIPS, GIGANTEA, LSH1, and FTSH varying by light quality (Tang et al., 2019). Gene Ontology enrichment revealed significant enrichment in xyloglucan:xyloglucosyl transferase activity and carbohydrate metabolic processes closely associated with light responses. The involvement of cell wall modification genes indicates that light quality affects not just photosynthesis but also growth through altered cell expansion, suggesting that LED-based protected cultivation systems could be optimized for both light spectrum and intensity to maximize yield.

### 6.6 Environmental effects on yield: transcriptional basis of genotype-by-environment interactions

Yield expression depends critically on environmental conditions, with transcriptomics revealing molecular mechanisms underlying genotype-by-environment (G×E) interactions. RNA-seq analysis of soilless substrate-based cultivation versus soil cultivation in tomato detected 476 DEGs, 321 upregulated, 155 downregulated, associated with 10.1% yield increase in soilless systems (Guo et al., 2022). This demonstrates that cultivation practices alter transcriptional programs governing growth and fruit quality, providing molecular explanations for agronomic observations and enabling prediction of which genotypes will respond favorably to specific management practices.

Under salinity stress, Affymetrix microarray analysis identified eight genes, JAZ8 (jasmonate signaling), Polcalcin jun (calcium binding), ER5, ATPase 3 (ion transport), XTH (cell wall modification), WRKY (transcription factor), and TSW12 showing significant positive correlations with total yield (Alsadon et al., 2015). These genes were recommended for incorporation into salinity-tolerant breeding programs, exemplifying how transcriptomics identifies expression-based markers predicting yield maintenance under stress a critical capability for breeding climate-resilient cultivars.

Fruit soluble solids and yield, both commercially important traits, result largely from fruit primary carbon metabolism (Causse et al., 2004). Microarray analysis of six introgression lines measuring Brix and yield revealed that known introgressed alleles altered expression of carbon metabolism enzymes at 20 days after anthesis (Baxter et al., 2005), demonstrating that yield-determining metabolic processes operate during specific developmental windows. This temporal specificity indicates that breeding for altered sugar content or yield requires considering not just which genes are present but when they are expressed during fruit development.

In tobacco, overexpression of GhKTI12 (a Kunitz trypsin inhibitor from cotton) enhanced seed yield and biomass. RNA-seq analysis of transgenic lines identified 518 upregulated and 406 downregulated genes common across overexpression lines (Myat et al., 2022). Most downregulated genes were MADS-box genes regulating flowering time—including MADS6, AP1, AP3, AGL8, AGL6, SEP1, and SEP2, while most upregulated genes governed cell division and differentiation, including RD21, TET8, KTN80, AOX1, AOX2, CP1, and KIC. This coordinated repression of flowering genes with simultaneous activation of cell division programs suggests that yield improvement resulted from extended vegetative growth and enhanced meristematic activity, demonstrating that engineering developmental timing can substantially increase biomass production.

Across diverse yield components and solanaceous species, transcriptomic studies reveal several actionable principles for breeding higher-yielding, climate-adapted cultivars. First, yield determination involves coordinated transcriptional networks spanning morphological development, metabolic regulation, hormone signaling, and stress responses not isolated single-gene effects. Master transcription factors like SlMX1 that regulate multiple yield-determining processes simultaneously represent high-leverage breeding targets enabling multi-trait improvement without negative genetic correlations.

Second, temporal dynamics are critical, developmental decisions determining final yield occur surprisingly early (potato tuber shape at stolon hook stage; carbon metabolism at 20 DAA in tomato fruit), necessitating phenotyping and transcriptome profiling across multiple developmental time points rather than focusing solely on mature organs. This temporal complexity also suggests that breeding for altered developmental timing, as demonstrated by GhKTI12 overexpression could substantially increase productivity by extending growth phases or optimizing source-sink relationships.

Third, yield under stress requires maintaining key physiological processes rather than just activating stress responses, nitrogen-use efficient eggplant maintains photosynthetic gene expression, salt-tolerant tomato maintains ion homeostasis genes, and parthenocarpic lines maintain auxin signaling. This indicates that breeding for yield stability should prioritize genotypes showing minimal transcriptional disruption of core metabolic processes under stress, rather than maximal stress-responsive gene activation.

Fourth, environmental factors (substrate type, light quality, CO_2_ levels, salinity) substantially alter yield-determining transcriptional programs, with cultivars showing differential transcriptional plasticity. Understanding these G×E interactions through multi-environment transcriptomics enables prediction of which genotypes will perform optimally under specific management practices or environmental conditions, essential for breeding cultivars adapted to specific production systems (protected cultivation, organic systems, saline soils) or future climate scenarios.

Fifth, epitranscriptomic regulation (RNA methylation) and cis-regulatory variation (ENO promoter) both contribute to yield variation, expanding breeding targets beyond traditional protein-coding genes. Genomic selection models can incorporate eQTL and regulatory variants alongside structural variants to capture the full spectrum of functional variation affecting yield.

Finally, the identification of expression-based markers showing consistent correlations with yield across environments (JAZ8, XTH, WRKY in tomato salinity study) provides practical tools for marker-assisted selection or genomic prediction, enabling early-generation selection on molecular profiles before yield itself can be accurately phenotyped. These transcriptomic insights collectively provide a molecular foundation for breeding solanaceous cultivars that achieve the genetic gain acceleration necessary to meet projected food demand while maintaining productivity under the increasingly variable environmental conditions imposed by climate change.

## 7 Conclusions and future prospects

### 7.1 Major achievements: from gene discovery to regulatory network elucidation

Transcriptomics has fundamentally transformed solanaceous crop improvement by revealing the molecular architecture underlying complex agronomic traits that have historically resisted genetic dissection. This review has synthesized progress across four major domains: (1) phytochemical biosynthesis controlling nutritional quality and flavor, (2) biotic stress resistance mechanisms spanning diverse pathogen types, (3) abiotic stress tolerance networks enabling climate adaptation, and (4) yield component determination integrating developmental, metabolic, and environmental factors. Several unifying principles emerge from this synthesis that have direct implications for accelerating genetic gain while addressing the consumer-producer trade-offs.

First, transcriptomic studies have consistently revealed that economically important traits whether capsaicinoid pungency, disease resistance, drought tolerance, or fruit size are governed not by single genes but by coordinated regulatory networks orchestrated by master transcription factors. The repeated identification of WRKY, MYB, bZIP, ERF, HSF, and NAC transcription factor families across all trait categories indicates these represent high-leverage breeding targets: manipulating single regulatory genes can trigger cascading expression changes across entire biosynthetic or defense pathways, potentially enabling multi-trait improvement without the negative genetic correlations that plague conventional phenotypic selection. For instance, SlMX1 in tomato simultaneously improves yield, trichome-mediated pest resistance, and stress tolerance, directly addressing the challenge of balancing production imperatives with consumer quality demands.

Second, temporal and spatial dynamics of gene expression are critical for trait manifestation but are frequently overlooked in breeding programs focused on mature phenotypes. Developmental decisions determining potato tuber shape occur at the stolon hook stage, capsaicinoid biosynthesis is restricted to placental tissue, and carbon metabolism during specific fruit development windows (20 days after anthesis in tomato) substantially influences final yield and quality. This temporal-spatial specificity implies that breeding strategies must incorporate stage-specific and tissue-specific transcriptome profiling to capture the full spectrum of functional variation, and that phenotypic selection at inappropriate developmental stages may miss causal genetic variation entirely.

Third, the discovery of regulatory RNAs (lncRNA16397 regulating SlGRX22 in P. infestans resistance; microRNAs modulating cold tolerance in potato), epigenetic modifications (DNA methylation affecting tomato ripening genes), epitranscriptomic regulation (RNA m6A modification improving potato yield), and cis-regulatory variation (ENO promoter mutation increasing tomato fruit size) reveals that breeding programs focused exclusively on protein-coding variation are accessing only a fraction of functional genetic diversity. Expression quantitative trait loci (eQTL) mapping and regulatory variant analysis should be systematically integrated into genomic selection models to capture this hidden genetic variation.

Fourth, transcriptomics has illuminated the molecular basis of genotype-by-environment interactions that confound breeding progress. Storage temperature profoundly affects tomato flavor volatile biosynthesis, nitrogen availability triggers distinct metabolic reprogramming strategies in efficient versus inefficient genotypes, and combined stresses elicit transcriptional responses unpredictable from single-stress treatments. This G×E understanding enables breeding strategies tailored to specific production environments—whether protected cultivation with controlled CO_2_ and light quality, organic systems with limited nitrogen inputs, or climate-stressed regions with co-occurring heat and drought.

### 7.2 Current limitations and knowledge gaps

Despite substantial progress, significant gaps constrain the translation of transcriptomic insights into breeding outcomes. First, the vast majority of studies employ bulk RNA-seq from heterogeneous tissues, masking cell-type-specific expression programs that may be functionally critical. For example, understanding why capsaicinoid biosynthesis is restricted to placental cells, or how vascular tissue mediates systemic stress signaling, requires single-cell or spatial transcriptomics approaches that are only beginning to be applied in solanaceous crops. The technical challenges of generating high-quality protoplasts from plants with thick cell walls have limited single-cell RNA-seq adoption, though emerging nuclei isolation protocols may circumvent this barrier.

Second, most transcriptomic studies are descriptive rather than functional, identifying differentially expressed genes without validating causal roles through reverse genetics. While candidate gene lists proliferate in the literature, relatively few have been functionally characterized through knockout, overexpression, or natural allelic variation studies. This validation bottleneck prevents confident prioritization of breeding targets and risks pursuing expression changes that are correlated with but not causal for desirable phenotypes. Integration of transcriptomics with genome editing technologies, particularly multiplex CRISPR approaches enabling simultaneous knockout of multiple candidates could accelerate functional validation.

Third, temporal resolution remains insufficient in most studies. Single-timepoint or coarse time-course analyses miss transient expression changes, oscillatory dynamics, and circadian-regulated processes that may be functionally important. High-resolution time-series experiments, while expensive, are essential for capturing the dynamic transcriptional responses underlying developmental transitions, stress acclimation, and fruit ripening all processes where timing critically determines outcomes.

Fourth, integration across omics layers, transcriptome, proteome, metabolome, epigenome remains limited despite universal recognition of its importance. Transcript abundance often correlates poorly with protein levels due to post-transcriptional and translational regulation, and metabolite concentrations reflect both biosynthesis and turnover rates that are not directly predictable from enzyme expression alone. The AsA inverse correlation between biosynthetic gene expression and accumulation, and the phenylalanine paradox where substrate accumulation doesn’t predict volatile production, exemplify why multi-omics integration is essential for accurate trait prediction and engineering.

Fifth, wild relatives remain underexploited despite harboring resistance and quality alleles absent from cultivated germplasm (Kaur et al., 2023; Malik et al., 2022). While several studies have examined wild eggplant (S. aculeatissimum, S. torvum) and identified massive transcriptional differences underlying superior stress tolerance or pest resistance, systematic transcriptomic characterization across wild Solanum diversity is lacking. Comprehensive wild germplasm transcriptome profiling could reveal novel biosynthetic pathways, regulatory mechanisms, and stress response networks unavailable in domesticated gene pools.

### 7.3 Emerging technologies and future directions

Several technological advances promise to address current limitations and accelerate trait discovery. Long-read sequencing platforms (PacBio Iso-Seq, Oxford Nanopore direct RNA sequencing) enable full-length transcript characterization, definitively resolving alternative splicing patterns, fusion transcripts, and allele-specific expression in heterozygous and polyploid genomes. Alternative splicing has been shown to generate protein isoforms with distinct or even antagonistic functions, yet remains poorly characterized in solanaceous crops. Comprehensive isoform catalogs for major cultivars and trait-relevant tissues would reveal previously invisible functional variation and enable isoform-specific marker development.

Spatial transcriptomics technologies, including Visium, Slide-seq, and emerging *in situ* sequencing methods preserve tissue architecture while profiling spatially resolved gene expression, enabling reconstruction of cell-type-specific expression programs without physical cell isolation. For solanaceous crops, spatial transcriptomics could illuminate: (1) gradients of gene expression during fruit ripening that determine quality trait distribution, (2) cell-type-specific pathogen responses revealing why certain tissues resist infection, (3) vascular-specific expression of nutrient transporters governing use efficiency, and (4) localized stress signaling networks determining whether damage remains contained or triggers whole-plant responses.

Multi-omics data integration through machine learning and network inference approaches can identify key regulatory nodes controlling complex traits. Co-expression network analysis has already identified hub genes regulating tomato flavor (Zhu et al., 2022), and extension to multi-layer networks integrating transcriptome-proteome-metabolome-phenome data would enable prediction of which genetic perturbations yield desired phenotypic outcomes. Such predictive models could guide gene editing targets, predict transgressive segregants in breeding populations, and identify optimal allelic combinations for genomic selection.

Pan-genome and structural variation (SV) analyses integrated with transcriptomics represent another frontier. A tomato pan-genome encompassing 238,490 SVs across 100 diverse lines demonstrated that structural variants, including presence/absence variations, copy number changes, and inversions, underlie fruit flavor, size, and yield variation (Alonge et al., 2020). Critically, an SV at the glycosyltransferase NSGT1 was shown to control phenylpropanoid volatile levels affecting flavor. SVs may frequently affect trait variation through altering gene expression (creating or disrupting regulatory elements) rather than protein function, making SV-eQTL mapping essential for comprehensive trait dissection. Moreover, SVs are often missed by short-read resequencing but readily detected by long-read sequencing, suggesting that current genomic selection models ignoring structural variation are capturing incomplete genetic architectures.

### 7.4 Translational applications: from transcriptome to improved cultivars

Realizing the breeding impact of transcriptomic discoveries requires systematic implementation in breeding programs, which remains limited. Expression-based markers, genes whose expression levels correlate consistently with trait values across environments could enable early-generation selection before phenotypes fully manifest. For example, identifying seedling-stage expression signatures predicting mature plant drought tolerance or fruit quality would dramatically accelerate breeding cycles. Developing robust expression-based prediction models requires large-scale, multi-environment transcriptome profiling of breeding populations, a substantial investment but potentially transformative for genetic gain rates.

Genomic selection models incorporating expression data alongside DNA sequence variation improve prediction accuracy for complex traits, particularly under novel environmental conditions not represented in training populations. eQTL mapping identifies variants affecting gene expression, which can be weighted in genomic prediction models based on biological priors (e.g., known pathway involvement). This functionally informed genomic selection could improve prediction of trait performance in untested environments or under climate scenarios not yet experienced, addressing the critical need for breeding cultivars adapted to future rather than historical climates.

Gene editing technologies, particularly base editing and prime editing enabling precise nucleotide changes coupled with transcriptomic identification of causal regulatory variants, enable direct engineering of desired expression patterns without creating transgenic plants. The ENO promoter mutation increasing tomato fruit size, or the SlAREB1 expression-enhancing variants conferring drought tolerance, could be engineered into elite backgrounds through non-transgenic editing, circumventing regulatory barriers to GMO deployment while leveraging transcriptomic insights.

Speed breeding protocols combined with transcriptome-guided selection could dramatically accelerate genetic gain. By profiling expression signatures at early growth stages under controlled stress treatments, breeders could select superior genotypes within months rather than years. This is particularly valuable for traits like multi-stress tolerance where field phenotyping requires multiple seasons and locations but transcriptional signatures might predict field performance from controlled environment tests.

### 7.5 Addressing the multi-trait breeding challenge

The introduction highlighted the critical challenge of simultaneously improving yield, stress resilience, and fruit quality traits, often showing negative genetic correlations when selected phenotypically. Transcriptomics has revealed why these correlations exist and how they might be broken. Stress tolerance often involves activating resource-expensive defense pathways (massive transcriptional reprogramming in V. dahliae-resistant eggplant) that divert resources from growth and fruit development. However, identification of genotypes maintaining yield under stress through minimal transcriptional disruption of core metabolic processes, rather than maximal stress response activation, suggests an alternative breeding strategy: selecting for stress resilience (maintaining function) rather than stress resistance (activating defenses).

Similarly, the inverse relationship between tomato refrigeration-induced flavor loss and shelf-life reflects transcriptional suppression of volatile biosynthesis at cold storage temperatures. Understanding this mechanistic basis enables targeted breeding: identifying or engineering cultivars that maintain volatile biosynthesis at low temperatures would simultaneously improve flavor and enable cold storage, breaking the apparent trade-off. Transcriptomics thus provides the mechanistic understanding necessary to design breeding strategies that overcome negative correlations through targeting underlying regulatory mechanisms rather than treating traits as independent.

Climate change imposes an urgent imperative to accelerate genetic gain in solanaceous crops while simultaneously improving stress resilience and maintaining quality attributes demanded by consumers and processors. Transcriptomics has transitioned from a gene discovery tool to a systems-level technology illuminating regulatory networks, developmental dynamics, environmental plasticity, and multi-trait integration. The path forward requires: (1) systematic application of emerging technologies (spatial transcriptomics, long-read sequencing) to address current knowledge gaps, (2) functional validation of candidate genes through genome editing, (3) multi-omics integration for predictive trait modeling, (4) large-scale expression profiling of breeding populations for genomic selection implementation, and (5) exploration of wild germplasm to access novel regulatory mechanisms.

Critically, realizing the breeding impact of transcriptomic insights demands closer collaboration between molecular biologists generating transcriptome data and breeders making selection decisions. Transcriptomic discoveries must be translated into actionable selection tools expression-based markers, eQTL-informed genomic selection, and guide RNA designs for precision editing. The substantial investment in transcriptome profiling made by the research community over the past two decades positions solanaceous crop breeding to meet future challenges, but only if these molecular insights are systematically integrated into breeding practice. The tools, knowledge, and opportunity exist; what remains is implementation to deliver the climate-resilient, high-quality, productive cultivars that global food security demands.
